# Patient Specific Characteristics Are an Important Factor That Determines the Risk of Acute Grade ≥ 2 Rectal Toxicity in Patients Treated for Prostate Cancer with IMRT and Daily Image Guidance Based on Implanted Gold Markers

**DOI:** 10.4172/2167-7964.1000225

**Published:** 2016-06-13

**Authors:** Xiaonan Liu, Jing Li, Teresa Wu, Steven E Schild, Michael H Schild, William Wong, Sujay Vora, Mirek Fatyga

**Affiliations:** 1 School of Computing, Informatics, Decision Systems Engineering, Arizona State University, Tempe Arizona, USA; 2 Department of Radiation Oncology, Mayo Clinic Arizona, Phoenix Arizona, USA

**Keywords:** Prostate cancer, Radiation therapy, IMRT, Acute rectal toxicity, Statins, PSA, Toxicity modeling, Logistic regression, LASSO

## Abstract

**Aim:**

To model acute rectal toxicity in Intensity Modulated Radiation Therapy (IMRT) for prostate cancer using dosimetry and patient specific characteristics.

**Methods:**

A database of 79 prostate cancer patients treated with image guided IMRT was used to fit parameters of Lyman-Kutcher-Burman (LKB) and logistic regression Normal Tissue Complications Probability (NTCP) models to acute grade ≥ 2 rectal toxicities. We used a univariate regression model to find the dosimetric index which was most correlated with toxicity and a multivariate logistic regression model with machine learning algorithm to integrate dosimetry with patient specific characteristics. We used Receiver Operating Characteristics (ROC) analysis and the area under the ROC curve (AUC) to quantify the predictive power of models.

**Results:**

Sixteen patients (20.3%) developed acute grade≥2 rectal toxicity. Our best estimate (95% confidence interval) of LKB model parameters for acute rectal toxicity are exponent n=0.13 (0.1–0.16), slope m=0.09 (0.08–0.11), and threshold dose TD50=56.8 (53.7–59.9) Gy. The best dosimetric indices in the univariate logistic regression NTCP model were D25% and V50Gy. The best AUC of dosimetry only modeling was 0.67 (0.54, 0.8). In the multivariate logistic regression two patient specific variables were particularly strongly correlated with acute rectal toxicity, the use of statin drugs and PSA level prior to IMRT, while two additional variables, age and diabetes were weakly correlated. The AUC of the logistic regression NTCP model improved to 0.88 (0.8, 0.96) when patient specific characteristics were included. In a group of 79 patients, 40 took Statins and 39 did not. Among patients who took statins, (4/40)=10% developed acute grade ≥2 rectal toxicity, compared to (12/39)=30.8% who did not take statins (p=0.03). The average and standard deviation of PSA distribution for patients with acute rectal toxicity was *PSA_tox_* = 5.77 ± 2.27 and it was *PSA_notox_* = 9.5 ± 7.8 for the remainder (p=0.01).

**Conclusions:**

Patient specific characteristics strongly influence the likelihood of acute grade ≥ 2 rectal toxicity in radiation therapy for prostate cancer.

## Core Tip

This is a retrospective study to evaluate methods of modeling of acute rectal toxicity in treatments of prostate cancer with intensity modulated radiation therapy, with particular emphasis on integrating dosimetry and patient specific characteristics. We found that two patient specific characteristics, use of statins and PSA levels, were dominant predictors of risk of acute rectal toxicity in this patient cohort. We found one prior report that statins had a protective effect against acute gastrointestinal toxicity in pelvic radiation therapy. We hypothesize that correlation with PSA was an effect of another medication which was not recorded in the study.

## Introduction

Accurate modeling of the likelihood of complications in radiation therapy is increasingly important due to recent trends to dose escalate and hypofractionate. A quantitative assessment of potential benefits of new treatment techniques is increasingly complex.

Because of low incidence of late toxicity in IMRT treatments [1] it is reasonable to expect that avoidance of acute toxicity will become more important in future clinical practice. For the same reason it may also be more practical to use acute toxicity as one of the tools with which to compare the efficacy and the therapeutic ratio of emergent treatment modalities [2]. To date very little work has been done on the modeling of acute rectal toxicity and the work that was done used 3D conformal techniques (3DCRT) [3]. Compared to late rectal toxicity, higher incidence of acute rectal toxicity means that relatively small databases of IMRT treatments can be used to obtain toxicity models. Our institution accumulated a database of 79 prostate patients who were treated exclusively with an IMRT technique and MRI directed boost dose to the region of the prostate with the greatest disease burden [4]. Each patient in this study had fiducial markers implanted into the prostate to improve the consistency of their daily setup. We observed 16 (20%) acute grade ≥ 2 rectal toxicity events. This number of acute toxicity events is sufficient to permit fitting of Normal Tissue Complications Probability (NTCP) models.

The first objective of this study was to establish a framework for modeling of acute rectal toxicity in image guided treatments with conformal delivery techniques. This specific need arose at our institution due to the opening of a proton pencil beam scanning facility. Existing models of acute rectal toxicity are based on data obtained with 3DCRT techniques with limited image guidance. Compared to these techniques image guided pencil beam scanning will produce more conformal dose distributions in the target which will also change dose distributions in organs at risk [5,6]. While not perfect, a model based on image guided IMRT technique should be a better approximation of toxicity rates in image guided pencil beam scanning than older models based on 3DCRT.

The second objective of this study was to incorporate patient specific characteristics into toxicity models. Understanding of the relationship between patient specific characteristics and toxicity can help in clinical practice but is also needed if one compares two treatment modalities through studies that were not acquired contemporaneously. An example would be comparing the present study to a future study of prostate patients who will be treated with pencil beam scanning at our institution. If the toxicity is affected by patient specific characteristics that do not significantly change over time such as age, genetics or comorbidities, studies can be compared on the basis of dosimetry alone. However, if the toxicity is affected by characteristics that do significantly change over time, such as the prevalence of use of common medicines, comparing studies on the basis of dosimetry alone could lead to misleading conclusions.

The Lyman-Kutcher-Burman (LKB) model has been commonly used in the past to model rectal toxicity [5]. We include this model in our study because it provides the best comparison to previously published studies. The LKB model is difficult to combine with patient specific characteristics however, because the log-likelihood function in the model is non-concave and thus difficult to fit when the size of a study is small. NTCP models which are based on logistic regression have more favorable fitting characteristics and were used in the past to study correlations between patient specific variables and late rectal toxicity [7]. We use a multivariate logistic regression NTCP model in this study but enhance it with two novel elements:

Firstly, we introduce a procedure which finds the optimum dosimetric index that can be used as a dosimetry correlate in the multivariate regression. The procedure is based on the univariate logistic regression NTCP model with a single dosimetric index and it consists of building a family of models which span a range of indices. The predictive power of each model is examined with Receiver Operating Characteristics (ROC) technique and the model which has the largest area under the ROC curve (AUC) is chosen as the best dosimetry correlate. The univariate logistic regression NTCP model can also be used as a dosimetry only model which provides a more direct relationship between a common dosimetric index and expected rates of toxicity. Such a model may be clinically more useful than the LKB model because a threshold on a dosimetric index is commonly used in treatment planning and in plan evaluation.

Secondly, we utilize a machine learning technique called the Least Absolute Shrinkage and Selection Operator (LASSO) [8] which automates the selection of correlated variables in multivariate logistic regression and is particularly effective when sample sizes are small.

## Materials and Methods

### Patients

This study included 79 patients who were treated for prostate cancer with Intensity-Modulated Radiation Therapy (IMRT) at Mayo Clinic Arizona between 2009 and 2012. The study was approved by the Institutional Review Board (IRB) of Mayo Clinic Arizona and included written informed consent from all subjects.

Median patient age was 75.7 years with a range 59.9–89.4. Nine patients had diabetes. The Gleason score ranged from 6 to 9 (median: 7). The PSA level prior to treatment ranged from 2.1 to 43 (median: 6.6). Sixteen (20.3%) of the 79 patients developed grade ≥ 2 acute rectal toxicity which were graded according to CTCAE v4 by a physician who retrospectively reviewed patient’s medical records. Three (3.8%) of the 79 patients developed grade ≥ 2 late rectal toxicity which were graded according to CTCAE v4 by a physician who retrospectively reviewed patient’s medical records. Statins were used by 40 of the 79 patients (50.6%). Metformin was used by 5 of the 79 patients (6.3%), alpha-blockers were used by 44 of the 79 patients (56%). Thirty three patients (41.8%) received hormonal treatment. The median follow up period was 31.7 months with a minimum of 4 months and a maximum of 57.4 months.

### IMRT treatment planning and delivery

A static field IMRT technique with 7 coplanar 6MV fields was employed. The whole prostate was designated as a clinical tumor volume (CTV), and two planning tumor volumes (PTV) were created using uniform 3mm and 6mm expansions. A dose of 77.4 Gy in 43 fractions (1.8Gy/fraction) was prescribed to the 3mm expansion, and a dose of 70Gy to the 6mm expansion. Seminal Vesicles with uniform 7mm expansion were prescribed 54Gy. A simultaneous integrated boost (SIB) was given to areas suspicious for cancer as demonstrated in a planning multi-parametric magnetic resonance scan which was a combination of T2-weighted imaging, Diffusion Weighted Imaging and Dynamic Contrast-Enhanced imaging [4]. The SIB volume was identified by a diagnostic radiologist specializing in genitourinary imaging, was not expanded, and was prescribed 81–83Gy. The rectum was drawn as a whole organ bounded by ischial tuberosity inferiorly and sigmoid flexure superiorly. All patients were planned using the Eclipse Treatment Planning System (TPS) produced by Varian, Inc. The dose was calculated on a 0.25cm × 0.25cm × 0.25cm rectangular grid using analytic anisotropic algorithm (AAA) in the Eclipse TPS. The maximum dose to the rectum was *D_max_* = 81.3 ± 1.2 *Gy*, the mean dose was *D_mean_* = 33.1 ± 5.7 *Gy*, the minimum dose to 10% of rectal volume was *D*_10%_ = 65.0 ± 5.9*Gy* and the minimum dose to 40% was *D*_40%_ = 34.6 ± 7.6*Gy*. Patients in the study had fiducial markers implanted into their prostates which were used for daily localization. Imaging for daily localization was done with MV techniques for early patients, transitioning to KV imaging techniques as these were introduced.

### Dosimetry data extraction

Dose Volume Histograms for all patients were extracted using automated scripts which were written within the Applications Programmer Interface (API) of Eclipse TPS. Dose volume information was written to disk and a custom software program using freely available “R” package [9] was used for the statistical analysis.

### Outline of data analysis

The analysis was divided into three distinct components: dosimetry only modeling, multivariate modeling with dosimetry and patient specific characteristics, and cross validation tests.

The dosimetry only modeling used data for all patients in the database and modeled correlations between treatment planning dosimetry and acute rectal toxicity without regard for patient specific characteristics. This modeling step had two purposes. Firstly, we used dosimetry only models to find the best representation of correlations with dosimetry that can be used in multivariate modeling with patient specific characteristics. Secondly, a comparison of dosimetry only modeling to the literature gave us confidence in toxicity assignments in our database. Toxicity assignments can be subjective, particularly in a retrospective study. Observing clear correlations between planning dosimetry and toxicities validated both toxicity assignments and the quality of clinical delivery of radiation treatments.

The multivariate modeling combines dosimetry with patient specific characteristics. We used multivariate logistic regression model to combine patient specific variables with dosimetric correlates which were identified in the dosimetry only modeling. In this step we used machine learning technique called Least Absolute Shrinkage and Selection Operator (LASSO) to automate the selection of most significant patient specific correlates.

Cross validation tests were used as additional tests of the validity of multivariate modeling. We modified the commonly used LKB and logistic regression models using one patient specific characteristic at a time to verify that results obtained through machine learning techniques could be reproduced by other methods. Our purpose was not only to validate this work, but also to build confidence that machine learning techniques are indeed applicable to the statistical analysis of clinical outcomes in radiation therapy.

### Normal Tissue Complication Probability (NTCP) modeling with dosimetry only

#### The Lyman-Kutcher-Burman (LKB) model

The LKB model [10,11] has been extensively used to model rectal toxicity in the past [5]. We used standard formulation of the model:

$$\begin{array}{l} \mathrm{NTCP}=\frac{1}{\sqrt{2\pi }}\int_{-\infty }^{t}e^{-\frac{\tau^{2}}{2}}d\tau \\ \mathrm{gEUD}={\left(\sum_{i}v_{i}D_{i}^{1/n}\right)}^{n}\;v\\ \mathrm{gEUD}={\left(\sum_{i}v_{i}D_{i}^{1/n}\right)}^{n} \end{array}$$

where *TD*_50_,*m*,*n* were adjustable parameters of the model. We used a Maximum Likelihood Estimation (MLE) technique and specifically the Nelder-Mead method [12] that has been implemented in the statistical software, “R” [13]. The asymptotic theorem of MLE [14] was used to compute error intervals.

#### Univariate Logistic Regression with dosimetry only

Univariate logistic regression was used to find the dosimetric index D which was most predictive for correlations between toxicity and dosimetry. We built a family of univariate models which span a range of indices and examined the predictive power of each model using the ROC analysis. An index which generates the highest AUC was used in multivariate analysis with patient specific characteristics. The univariate model is formulated as follows:

$$\log\left(\frac{\mathrm{NTCP}}{1-\mathrm{NTCP}}\right)=\propto_{0}+\gamma D$$

where *D* is a standard dosimetric variable such as *V*%*_D_*, which is a volume fraction that receives a dose D or greater, or *D_X_*_%_ which is the lowest dose received by the volume fraction *X*% and parameters ∝_0_ and *γ* are parameters which are estimated by MLE.

### Normal Tissue Complication Probability (NTCP) modeling with dosimetry and patient specific characteristics

#### Multivariate logistic regression NTCP model

An NTCP model based on logistic regression [7] was used in a relatively recent works by Cella et al. [15] and by Lee et al. [16]. The advantage of such a model is that its log-likelihood function is concave which facilitates multivariate fitting, even with limited statistics. The model is formulated as follows:

$$\log\left(\frac{\mathrm{NTCP}}{1-\mathrm{NTCP}}\right)=\alpha_{0}+\sum_{k=1}^{p}\alpha_{k}x_{k}+\gamma D$$

*x*_1_,…,*x_p_* are patient characteristic variables and *D* is a standard dosimetric variable such as *V*%*_D_*, which is a volume fraction that receives a dose D or greater, or *D_X_*_%_ which is the lowest dose received by the volume fraction *X*%. Parameters *α*_0_, *α*_1_,…*α_p_*, *γ* are estimated by MLE. Patient characteristic variables can be categorical or continuous. Categorical variables assume a value of 0 or 1. For example, the use of statins is assigned a value of 1 if a patient is a statin user, and a value of 0 if a patient is not. Continuous variables, like age or PSA level, assume the value which is reported for a particular patient.

We employed the Least Absolute Shrinkage and Selection Operator (LASSO) [8] to automate the selection of patient specific variables included in the final logistic regression fit. LASSO is a well-established machine learning method that selects a small subset of significant predictors from all the predictors included in the model. It is especially useful when one wants to produce a robust model with a small sample size. The LASSO operator is described in greater details in the Appendix (VI.2).

#### Patient specific characteristics

We examined the following variables: age, diabetes, hormonal treatment (stratified as neoadjuvant/ concurrent/adjuvant), use of statins, use of metformin, use of alpha-blockers, whole prostate volume, MRI boost volume, rectal volume, PSA prior to IMRT, and Gleason Score.

Two of the variables, age and diabetes have been reported to be associated with late rectal toxicity [17–20]. Volume contouring could have been associated with systematic biases in the dosimetry. Statin use was previously reported as protective against acute gastrointestinal toxicity [21]. Remaining variables were not previously reported as risk factors. Since this was a retrospective study, we were not able to examine all variables that were previously reported as correlated with late rectal toxicity, most notably cardiovascular disease and prior abdominal surgery.

#### Sample Size

We used standard sample size tables for logistic regression [22] to verify that our sample size is large enough for the study. Assuming α error of 0.05 and the effect size (odds ratio) of 2.5, with the prevalence of acute rectal toxicity being 20%, the sample size should be larger than 73 in order to achieve a >70% power of differentiating patients with and without toxicity. Parameters chosen for this estimation are often used in medical studies hence the sample size of 79 patients meets the criteria which are typically set for medical studies.

### Cross validation tests

Because of relatively low statistics in this data sample we performed a number of cross validation tests to confirm the validity of our results. These tests fall into two broad categories: standard statistical tests and modified or partial models. Partial models refer to LKB and logistic regression models in which we used the dosimetric predictor and one patient specific variable at a time. Using one patient specific variable at a time is necessary in the LKB model because of adverse numerical characteristics of the model which preclude its extension to a larger number of patient specific variables on a small data sample. Using partial models provides a cross check on the performance of the LASSO operator and also provides clinically useful estimates of acute rectal toxicity rates in situations when all of the variables which were used in this work are not available. Details of cross validation tests are provided in the Appendix VI.3.

### Dose conversion

In accordance with QUANTEC report $\frac{\propto }{\beta }$ [5] physical doses were converted to a 2Gy dose equivalent using *β* ratio of 3Gy. We used a voxel-by-voxel correction which corrects each voxel that belongs to a structure to a 2Gy equivalent dose. The dose in each voxel is computed by trilinear interpolation from the dose matrix. This method of correction is similar but more precise than the bin-by-bin correction of the DVH which is typically recommended in the literature [23]. Final, corrected DVH is built on the basis of corrected doses in voxels. Voxel size corresponds to the resolution of the CT image which was typically 0.1cm × 0.1cm × 0.25cm.

## Results

### Dosimetry only modeling

Sixteen (20.3%) patients developed grade ≥ 2 acute rectal toxicity.

#### LKB model

The estimated LKB parameters are summarized in Table 1, top row. The parameters ‘m’ and ‘n’ of the fit are similar to the LKB formula published by the QUANTEC group which modeled late rectal toxicity [5]. To quantify this apparent similarity we performed the ROC analysis of QUANTEC late rectal toxicity formula using acute rectal toxicity data which yielded an AUC=0.67, comparable to the AUC of our own fit (Table 1, bottom row). The incidence of toxicity predicted by the QUANTEC formula (3.5%) is much lower than the observed acute rectal toxicity (20%), but the QUANTEC model remains predictive for relative risk within our patient cohort.

#### Univariate logistic regression

**Figure 1:**
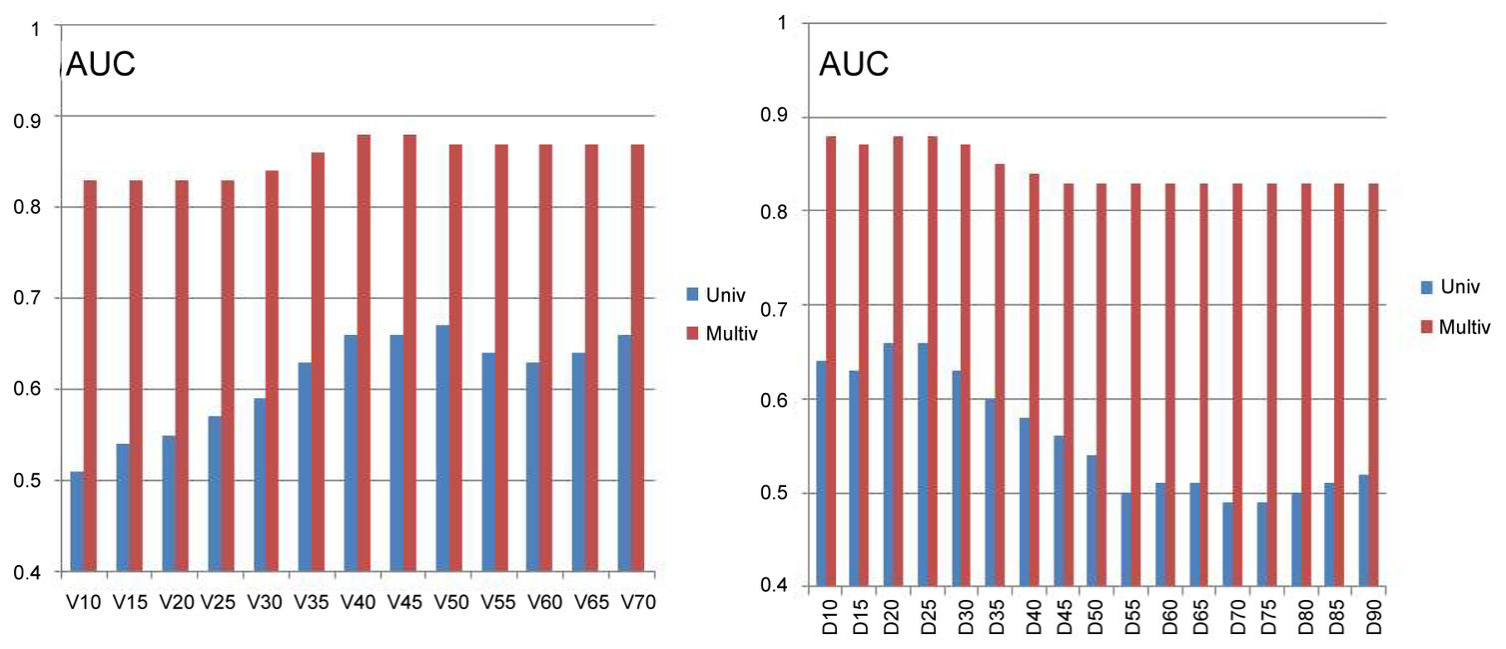
Summary of AUC values obtained for univariate logistic regression fits using a range of dosimetric indices, and multivariate logistic regression fits using the corresponding dosimetric index and patient specific variables. Right panel shows results using *D_X_*_%_ index (X% of the volume receives dose D or greater), and the left panel shows *V*%*_D_* index (volume fraction which receives dose equal or greater than D). Both panels show that best predictors of rectal toxicities are moderate to high doses applied to small volumes. The AUC of the model is significantly increased by adding patient specific variables. It can also be seen that a model which includes patient specific variables remains predictive even if the dosimetric index is not predictive at all.

Univariate logistic regression was performed using two DVH indices. *D_X_*_%_ is the dose such that *X*% of rectal volume receives this dose level or higher, *V*%*_D_* is the percentage of rectal volume which receives dose D or greater. Multiple univariate logistic regressions were performed within a range of values of X%=(10%–95%) and a dose D=10Gy–70Gy, with results summarized in Figure 1 (blue bars). Indices which maximize the AUC of the model are *D*_25%_ and *V*%_50_ and the maximum AUC for a dosimetry only fit is equal to 0.66.

### Acute toxicity with dosimetry and patient specific variables

#### Multivariate Logistic Regression

Multivariate logistic regression included one dosimetric index (*D*_25%_ or *D*_25%_) and all available patient specific variables. The LASSO operator was used to automatically select patient specific variables that were predictive for acute rectal toxicity. Following four variables were selected by the LASSO operator as significant: age, diabetes, PSA prior to IMRT, and use of statins. Fit parameters obtained in regression runs using *D*_25%_ and *V*%_50_*_Gy_* as dosimetric correlates are shown in Table 2 and in Table 3. Results of three independent fits are shown: a fit which includes all four variables selected by LASSO, a fit which includes only the two most strongly correlated variables PSA and statins, and a univariate fit using the dosimetric index alone. One notes that diabetes and age are not statistically significant in the full fit in spite of being selected by the LASSO operator, which could occur if these two variables are correlated. In Figure 1 (red bars) we show AUC of a family of multivariate logistic regression models, each using a different dosimetric index.

#### Data underlying most significant correlations

Unprocessed data underlying the two strongest correlation results can be summarized as follows:

In a group of 79 patients, 40 took Statins and 39 did not. Among patients who took statins, (4/40)=10% developed acute grade ≥ 2 rectal toxicity, compared to (12/39)=30.7% who did not take statins. Use of statins is a statistically significant independent predictor of acute rectal toxicity (Appendix VI.3.3).

The average and standard deviation of PSA distribution for patients with acute rectal toxicity is *PSA_tox_* = 5.77 ± 2.27 and it is *PSA_notox_* = 9.5 ± 7.8 for the remainder. The difference is statistically significant (Appendix VI.3.3).

By combining the use of statins and the PSA level one obtains a statistically significant predictor of acute rectal toxicity in multivariate logistic regression model with or without dosimetry. This is shown in Figure 1 which presents AUC of a multivariate logistic regression model as a function of a dosimetric index used in the fit. The AUC of the multivariate model is never lower than 0.8 (red bars) even when the dosimetric index is not predictive in univariate analysis (blue bars).

## Discussion

### NTCP models based on dosimetry alone

The fit of the LKB model yielded AUC=0.67 which falls within the range of values that are typically found in the literature for dosimetry only models of late rectal toxicity [5,7,23,24]. A comparison of our LKB fit to the QUANTEC model of late rectal toxicity strongly suggests that risks of acute and of late rectal toxicity depend on similar dose-volume variables, and that the main difference between these two types of toxicity is the threshold dose. To confirm this similarity we applied the QUANTEC model to acute rectal toxicity data and performed ROC analysis which yielded AUC=0.67, the same as our own fit. The QUANTEC model predicts absolute incidence of toxicity (3.5%) which is much lower than the observed incidence of acute toxicity (20%), but it predicts relative risk of acute toxicity in our patient cohort as well as our own fit. These observations are further supported by the results of univariate logistic regression analysis (Figure 1, blue bars), showing that the best predictors for acute toxicity are medium to high doses applied to small volumes, which is equivalent to parameter ‘n’ in the LKB fit being less than one.

Results of our study disagree with prior modeling of acute rectal toxicity using 3DCRT data [3] which concluded that mean rectal dose was the best predictor of acute rectal toxicity. The disagreement between this study and prior findings could be caused by differences in dose distributions but it could also be caused by a better correlation between planned and delivered doses when patients are treated with daily image guidance based on marker seeds implanted into the prostate. One notes that the incidence of acute rectal toxicity in our study was similar to the incidence of late rectal toxicity in earlier studies which were based on 3DCRT [5], while the incidence of late toxicity in our study was approximately five times lower. It is plausible that one needs to achieve good correlation between planned and clinically delivered dosimetry to observe true dependence of acute rectal toxicity on rectal dosimetry.

The univariate logistic regression model with a single dosimetric index achieves maximum AUC which is essentially the same as the AUC of the LKB model. This means that a single dosimetric index can be used as a dosimetry correlate in the multivariate logistic regression model without a loss of generality. One should stress that our result does not prove that a univariate model performs as well as the LKB model when extrapolated to significantly different rectal dosimetry that could be obtained with different delivery modalities. It does show however that a single dosimetric index is as good a predictor of the relative risk of acute rectal toxicity as the LKB model, when applied to a cohort of patients who were treated with the same IMRT technique and a daily image guidance.

**Figure 2:**
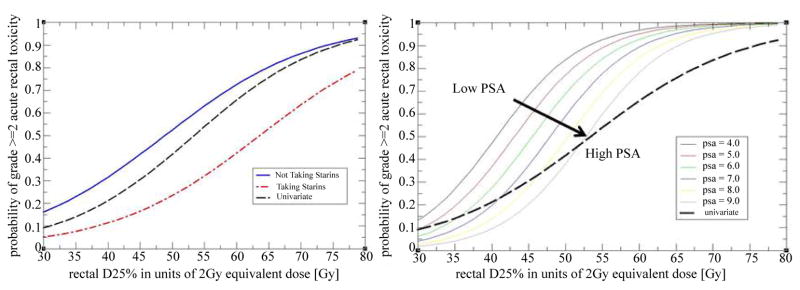
NTCP predictions of logistic regression models shown in in Table 4 which use a single dosimetric variable *D*_25%_ and a single patient specific variable. The upper panel (2a) shows the effect of Statin use while the lower panel (2b) shows the effect of the PSA level. Predictions of the univariate logistic regression fit with *D*_25%_ are shown in both panels by a black dashed curve.

The univariate logistic regression model can be used in clinical practice more directly than the LKB model. This is illustrated in Figure 2 where a dashed black curve represents a relationship between likelihood of toxicity and a value of the dosimetric index. A clinician can use Figure 2 to set a threshold on the dosimetric index which corresponds to the maximum acceptable incidence of toxicity, or read off the expected likelihood of toxicity for a given value of the dosimetric index in a treatment plan. Since standard dosimetric indices are computed by all treatment planning systems, Figure 2 could be used as a nomogram without a need for additional software implementation.

### Including patient specific characteristics in the NTCP model

#### Observed correlations

We found four patient specific characteristics (variables) which appear correlated with acute rectal toxicity: age, diabetes, statin use, and PSA prior to treatment. Two of the correlations (Statins and PSA) were strong enough to be classified as independent predictors of toxicity competing in correlation strength with dosimetry, within the range of dosimetric variability in this study. A relatively weak correlation between age [19], diabetes [17,18,20] and late rectal toxicity has been previously reported. The same correlation in the present study of acute rectal toxicity is also relatively weak.

The use of statins appeared protective against acute gastrointestinal toxicity, and the impact on NTCP was very significant. A patient who used statins was approximately three times less likely to develop acute rectal toxicity than a patient who did not. The magnitude of the effect is illustrated in Figure 2a where we plot predictions of the model stratified by statin use. A search of the PubMed database revealed one recent study [21] which reported that use of statins was protective against acute gastrointestinal toxicity during pelvic radiation therapy. The endpoint of the study was a bowel disease questionnaire and the data were not stratified by the disease site. We nonetheless consider both findings to be mutually corroborating. Results of recently concluded Phase II clinical trial suggest that statins may also have a protective effect against late rectal toxicity [24–26] and possible biological mechanisms have been investigated [27]. Our result can thus be seen as adding to the growing body of evidence that the use of statins may have a protective effect against rectal toxicity in radiation therapy and that the protective effect may be quite pronounced, at least in some cases. We did not have more specific data on exact statin and dosages used to further investigate this correlation. One should be cautious extrapolating this study to different patient populations and treat our finding as provocative rather than definitive.

Patients who developed acute rectal toxicity had, on average, a lower PSA prior to treatment than those who did not. Model predicts that a change in PSA by 1 ng/mL changes NTCP by a factor of approximately 1.5. The magnitude of the effect is shown in Figure 2b where predicted NTCP is stratified by the PSA level. A search of the Pubmed database did not find any prior studies reporting a similar effect. However, the same search revealed recent studies [28–31] which reported that men who used many commonly prescribed medicines had significantly reduced PSA levels. Examples of medicines which were associated with lower PSA level are: aspirin (−4%,−45%), statins (−4.6%,−13%), metformin (−14%), insulin (−16%), NSAIDs (−6%), and thiazide diuretics (−26%). One of the studies [29] reported evidence of a compounding effect when statins and thiazide diuretics were used together (−36%). These observations led us to a hypothesis that the correlation between reduced PSA level and acute rectal toxicity may be an indication that some patients in our study used medicines which, alone or in combination, caused two simultaneous side effects of reducing their PSA level and of increasing their susceptibility to radiation injury. As a further support for this hypothesis one notes that adding PSA level to the model increases the slope of response and decreases the threshold dose when compared to a univariate analysis (Figure 2b). This behavior is consistent with an interpretation that a hidden factor, an unrecorded use of a medicine, increased the susceptibility of some patients to radiation injury.

We considered other explanations for the observed correlation between PSA level and acute rectal toxicity, but could not find a convincing alternative. High PSA could be associated with a more aggressive treatment which could in turn increase the risk of toxicity, but the opposite is being observed. PSA levels could be correlated with the size of the prostate, but the size of the prostate does not correlate with toxicity. Hormonal treatment could affect PSA level, but hormonal treatment is not correlated with toxicity. Finally, there is no convincing biological explanation why higher PSA would, by itself, be protective against acute rectal toxicity.

#### Use of common medicines should be included in the analysis of acute rectal toxicity data

In this study we observed strong correlations between acute rectal toxicity and two patient specific characteristics, use of statins and PSA level prior to treatment. Incorporating both into an NTCP model raises the AUC of the model to a high value of 0.86. Both of these correlations suggest, directly and by inference, that the likelihood of acute rectal toxicity in an individual patient can be strongly affected by commonly used medicines. These effects may be strong enough to affect our ability to compare the efficacy of evolving treatment techniques. As an example, our study suggests that patients who took statins experienced a threefold reduction in the risk of acute rectal toxicity. During the decade of 2000–2010, which coincided with the broad introduction of IMRT into clinical practice, the prevalence of the use of statins in older men increased from 20% to 50% [32]. If all other factors were kept constant, one would expect that the incidence of acute rectal toxicity in radiation therapy for prostate cancer would have declined during this decade by 23% (factor of 0.77) solely because of changes in the prevalence of use of statins. Statins represent only one of many medicines that are used by older patients today. Data on use of prescription medicines in the US [33] shows that in years 2008–2009 64% of those older than sixty years of age used three or more medicines, while 37% used five or more. One therefore needs to consider not only the effects of individual medicines but also of their interactions. One should finally note that the impacts of common medicines on toxicity of radiation therapy are unintended side effects which may not persist in new generations of medicines. This raises a possibility that the incidence of toxicities could unexpectedly increase in future treatments in spite of improvements in treatment delivery techniques [34,35].

In summary, our study suggests that future studies of toxicities in radiation therapy need to collect data on the use of medicines by study patients and to include this information in the statistical analysis of treatment outcomes. Retrospective studies of toxicities which include data collected over long periods of time should consider patterns of use of common medicines as a potential confounding factor.

## Conclusion

We developed a framework for modeling of acute rectal toxicity in prostate treatments which integrates dosimetry with patient specific characteristics. We applied the approach to a database of 79 prostate patients treated with image guided IMRT technique. Results show that the likelihood of grade >= 2 acute rectal toxicity depends on intermediate to high doses delivered to small volumes which is qualitatively similar to late rectal toxicity. Observed correlations with patient specific characteristics suggest both directly and by inference that commonly used medicines can significantly influence the incidence of acute rectal toxicity in radiation therapy for prostate cancer. We suggest that future studies of toxicity would benefit from collecting data on patient’s use of common medicines.

## Limitations of This Study

This is a retrospective study with limited statistics. Some commonly studied patient specific variables could not be included in the study as they were not available.

## Supplementary Material

Appendix

## Figures and Tables

**Table 1:** Parameters and 95% confidence intervals of the LKB model using dosimetry only and describing grade ≥2 acute rectal toxicity. QUANTEC late rectal toxicity model is shown for comparison.

	*TD*_50_	*m*	*n*	AUC
Acute Toxicity	56.8 [53.7, 59.9]	0.093 [0.077, 0.108]	0.131 [0.099, 0.163]	0.67 [0.54, 0.80]
QUANTEC	76.9 [73.7, 80.1]	0.13 [0.10, 0.17]	0.09 [0.04, 0.14]	0.67 [0.54, 0.81]

**Table 2:** Parameters of logistic regression fits to grade ≥ 2 acute rectal toxicity using dosimetric index *D*_25%_. Error ranges are at 95% confidence level intervals.

3	$\log\left(\frac{\mathrm{NTCP}}{1-\mathrm{NTCP}}\right)\sim D_{25\%}$, age, diabetes, PSA, statins Multivariate	$\log\left(\frac{\mathrm{NTCP}}{1-\mathrm{NTCP}}\right)\sim$, PSA, statins Multivariate	$\log\left(\frac{\mathrm{NTCP}}{1-\mathrm{NTCP}}\right)\sim D_{25\%}$ Univariate
*α*_0_	−9.1 [−20.50, 0.16] P=0.08	−4.5 [−10.3, 0.41] P=0.1	−5.23 [−9.93, −1.40] P=0.015
*D*_25%_	0.19 [0.04, 0.39] P=0.03	0.19 [0.048, 0.364] P=0.02	0.098 [0.002, 0.212] P=0.064
PSA	−0.53 [−0.99, −0.20] P=0.008	−0.53 [−0.99, −0.20] P=0.009	Not Applicable
Statins	−2.03 [−3.79, −0.57] P=0.01	−1.83 [−3.40, −0.49] P=0.01	Not Applicable
Age	0.056 [−0.051, 0.176] P=0.32	Not Applicable	Not Applicable
Diabetes	1.73 [−0.52, 4.13] P=0.13	Not Applicable	Not Applicable
AUC	0.88 [0.80, 0.96]	0.86 [0.78, 0.95]	0.66 [0.49, 0.76]

**Table 3:** Parameters of logistic regression fits to grade ≥ 2 acute rectal toxicity using a dosimetric index *V*%_50_*_Gy_*. Error ranges are at 95% confidence level intervals.

	$\log\left(\frac{\mathrm{NTCP}}{1-\mathrm{NTCP}}\right)\sim V{\%}_{50}$, age, diabetes, PSA, statins Multivariate	$\log\left(\frac{\mathrm{NTCP}}{1-\mathrm{NTCP}}\right)\sim V{\%}_{50}$, age, PSA, statins Multivariate	$\log\left(\frac{\mathrm{NTCP}}{1-\mathrm{NTCP}}\right)\sim V{\%}_{50}$ Univariate
*α*_0_	−5.80 [−15.62, 2.55] P=0.20	−1.30 [−4.53, 1.69] P=0.4	−3.76 [−6.60, −1.35] P=0.004
*V*%_50_*_Gy_*	23.42 [3.98, 46.80] P=0.03	24.39 [6.19, 46.21] P=0.01	14.72 [0.31, 30.86] P=0.05
PSA	−0.48 [−0.91, −0.18] P=0.008	−0.49 [−0.91, −0.20] P=0.007	Not Applicable
Statins	−1.87 [−3.61, −0.43] P=0.01	−1.70 [−3.24, −0.37] P=0.018	Not Applicable
Age	0.06 [−0.05, 0.18] P=0.30	Not Applicable	Not Applicable
Diabetes	1.46 [−0.86, 3.87] P=0.21	Not Applicable	Not Applicable
AUC	0.87 [0.78, 0.96]	0.86 [0.77, 0.95]	0.67 [0.53, 0.80]
