# Supplementary material for: Patient Specific Characteristics Are an Important Factor That Determines the Risk of Acute Grade ≥ 2 Rectal Toxicity in Patients Treated for Prostate Cancer with IMRT and Daily Image Guidance Based on Implanted Gold Markers
Source: OMICS J Radiol. Author manuscript; Available in PMC 2016 Jul 29. (PMC4966533; doi:10.4172/2167-7964.1000225)
Supplement: Appendix [file NIHMS798547-supplement-Appendix.pdf]

13. APPENDIX

Statistical Methods

VI.1 Maximum Likelihood Fitting

To estimate model parameters we use the Maximum Likelihood Estimation (MLE) method with the following likelihood function:

$$l = \sum_{j=1}^{N} \left\{ y_j logNTCP_j + \left(1 - y_j\right) log\left(1 - NTCP_j\right) \right\}$$

where y_j is the toxicity indicator for patient j which assumes a value of 0 for patients who do not experience grade ≥ 2 rectal toxicity and the value of 1 for those who do, and $NTCP_j$ is a probability of a toxicity event computed for a given parameter set. The maximum likelihood fitting is accomplished using the freely available "R" package [13], and specifically the "MaxLik" [9] function. To fit the LKB model in the R environment, we used maxLik(l, start=c(65,0.2,0.3), method="nm") where "l" is the likelihood function of the LKB model, (65,0.2,0.3) are initial values for three parameters of the LKB model (TD50, m, n, respectively), and "nm" stands for the Nelder-Mead method [12]. After being executed, this function returns an MLE estimate for parameters $TD_{50}, m,$ n . "maxLik" was also used for the modified LKB model with one additional parameter.

To fit the logistic regression we used the same functional form of the likelihood function but replaced the "nm" function with "glm" function in R. To invoke the LASSO operator in multivariate logistic regression we used the "glmnet" function.

VI.2 Selection of significant predictors in Logistic Regression by LASSO

There are many patient-specific variables that may be potentially associated with the risk of toxicity. We have therefore used a machine learning method to automatically select the significant predictors among the set of potentially significant predictors. LASSO is a machine learning method developed in 1996 to specifically serve this purpose. Since being published, the LASSO paper [8] has been cited over 12,000 times and became one of the most popular techniques of the kind. LASSO is known to outperform competing methods especially when the sample size of the study is limited.

LASSO works by adding a constraint on the coefficients of the logistic regression, $\alpha_1, \ldots, \alpha_p, \gamma$, during the MLE estimation. That is, LASSO aims to find an MLE estimate for the coefficients, but subject to a constraint that the sum of the absolute values of the coefficients cannot exceed a pre-set threshold t, i.e., $\sum_{k=1}^{p} |\alpha_k| + |\gamma| \le t$. This constraint is known as the L1-penalty. L1-penalty has an advantageous mathematical property that, with a properly set threshold t it can guarantee the estimates for insignificant coefficients to be exactly zero, thereby selecting out the insignificant predictors. The remaining predictors are significant ones in terms of predicting the toxicity. An optimum value of the threshold t can be found by data-driven procedures like cross validation. In the present

analysis we used 10-fold cross validation with deviance as a tool to select the most appropriate value of the threshold. The procedure is implemented in the R package by a function “cv.glmnet” in the “glmnet” package.
We applied LASSO to an initial set of nine patient-specific variables plus a single dosimetric variable. LASSO selected age, diabetes, use of Statin drugs, PSA, and the dosimetric variable as significant predictors.

VI.3 Additional testing

The number of patients in our database is lower than in a typical study used by QUANTEC meta-analysis (Table 1 in [5]). The smallest QUANTEC study had 128 patients (84 after stratification), the largest study had 1024 patients, and a typical study had approximately 450 patients. Since the incidence of acute rectal toxicity in our study is only slightly higher than the incidence of late rectal toxicity in studies chosen by QUANTEC, the number of toxicity events in our study is lower as well. The number of toxicity events in QUANTEC studies falls between 9 and 138, with most studies having approximately 30 events after stratifications. To ensure the validity of our results we performed a sequence of additional statistical tests of stability and generalizability of our results, as follows:

VI.3.1 Statistical oversampling test in the LKB model

Since the LKB model has unfavorable numerical characteristics we tested the robustness of the dosimetry only LKB fit (Table 1) by the statistical oversampling technique. Parameters of the fit remain within their error intervals while the minority class is doubled, tripled, and quadrupled.

VI.3.2 Test of dose-volume dependence in the dosimetry only LKB model

It has been previously reported [3] that acute rectal toxicity in patients treated with 3DCRT technique may depend predominantly on mean rectal dose. Our analysis indicates that acute rectal toxicity in IMRT treatments depends primarily on intermediate to high doses, not mean rectal dose. The conclusion in the present work is supported by three independent observations: our own LKB fit (Table 1), the univariate logistic regression analysis (Figure 2, Table 2, Table 3), and the ROC analysis of QUANTEC formula (Table 1). Because of the discrepancy with prior work we performed one additional test of the robustness of the conclusion by fitting the LKB model to acute toxicity data with GEUD exponent (parameter ‘n’, Table 1) set to $n=1$. The ROC analysis of the new fit showed that the AUC of the model decreased significantly, from 0.67 in the unconstrained fit to 0.56 in the constrained fit, and the confidence interval no longer excluded 0.5. One therefore concludes that $n < 1$ is favored by the LKB fit.

VI.3.3 Statin use and PSA level as independent predictors of toxicity

To test if the use of Statins is an independent predictor of acute rectal toxicity we performed a univariate logistic regression fit using Statins as a sole variable. Both parameters of the fit are statistically significant with $p=0.02$ (intercept) and $p=0.03$ (coefficient) which shows that Statin use alone is an independent predictor of acute rectal toxicity.

The average and standard deviation of PSA distribution for patients with acute rectal toxicity is $PSA_{tox} = 5.77 \pm 2.27$ and it is $PSA_{notox} = 9.5 \pm 7.8$ for the remainder. To test the statistical significance of this difference we performed a Wilcoxon rank sum test (a non-parametric version of the two-sample t test, because PSA data does not follow normal distributions). The test yielded a p value of 0.01, indicating that the PSA for patients with grade ≥ 2 acute toxicity is significantly lower than that for the remainder.

VI.3.4 Cross checking the LASSO operator using modified LKB and restricted logistic regression models

Since the LASSO operator has not been used in similar toxicity studies before we tested the performance of LASSO against modified LKB and restricted multivariate logistic regression models.

LKB model has been modified with patient specific characteristics in past studies [34]. We had to limit the modified LKB model to one patient specific variable at a time because the non-concave log-likelihood function in the model makes fitting numerically intensive and including more than one variable may prevent convergence to a global maximum. The modified LKB model (mLKB), re-parameterizes TD_{50} to be patient specific,

$$TD_{50} = \beta_0 + \beta_1 x$$

where x is a patient specific variable which is one of the same variables that were used in multivariate logistic regression, and β_0, β_1 are parameters to be estimated. We chose to modify TD_{50} because in the model it quantifies the general susceptibility of a given patient to complications during radiation therapy, and thereby is most likely to be patient-specific. Parameters n and m describe the dose-volume dependence of NTCP and the slope of dose response, and therefore are more likely to depend on the organ architecture than on patient-specific characteristics. We consider a patient specific variable to be significantly correlated with toxicity if the AUC of the model increases with respect to the dosimetry only baseline and if the confidence interval for parameter β_1 excludes zero. To cross check the LASSO operator we ask if every variable selected by LASSO also shows significant correlation in mLKB, and whether variables that were not selected fail the correlation criteria. Results of testing with the mLKB model are shown in Table 4 which only shows patient specific variables that met the significance criteria above. Findings by the LASSO operator qualitatively agreed with a cross check against the modified LKB model.

Restricted logistic regression is defined in this work as a logistic regression model which includes the dosimetric index and one patient specific variable at a time. We ask whether results obtained through this model agree with mLKB and selections made by the LASSO operator. We consider the correlation with patient specific variable to be significant if the AUC of the model increases significantly from the dosimetry only baseline and the correlation coefficient is statistically significant. Results of testing with the restricted multivariate logistic regression model are shown in Table 5. Findings by the LASSO operator qualitatively agreed with a cross check against the restricted multivariate regression model. In Figure 2 we graph models which include statin use only (Figure 2a) and PSA level (Figure 2b)

VI.3.5 Leave One Out Cross Validation

Leave one out cross validation is a model validation technique which is particularly useful when data sets are too small to be divided into training and validation data sets [35]. We used this method to validate the logistic regression model which uses one dosimetric variable, Statin use and PSA level (Tables 2 and 3). The AUC of the model decreased from 0.86 to 0.81 for $D_{25\%}$ + Statins + PSA ,and from 0.86 to 0.80 for V_{50Gy} + Statins + PSA. A decrease in AUC is expected but the model remains highly predictive, hence, the test indicates good generalization capability of the model.

VI.3.6 ROC Curves

Example of ROC curves for dosimetry only models, multivariate logistic regression model, and modified LKB (mLKB) model are shown in Figure 3.

Table 4: Parameters and 95% confidence intervals of the modified LKB model (mLKB) describing grade ≥2 acute rectal acute toxicity.

Patient characteristic variables	β_0	β_1	m	n	AUC
Diabetes	55.78 [53.26,58.30]	-3.14 [-3.8,-2.48]	0.093 [0.080,0.106]	0.144 [0.112,0.172]	0.68 [0.55, 0.81]
Age	68.82 [65.08,72.57]	-0.16 [-0.21, -0.12]	0.096 [0.08,0.112]	0.134 [0.103,0.165]	0.69 [0.56, 0.83]
PSA	50.00 [47.1,52.9]	0.625 [0.54,0.71]	0.068 [0.061,0.074]	0.142 [0.129,0.168]	0.79 [0.69, 0.91]
Statins	63.32 [60.96,65.67]	3.47 [3.0, 3.95]	0.058 [0.049,0.067]	0.062 [0.047,0.077]	0.74 [0.64, 0.85]

Table 5: Parameters of multivariate logistic regression fit to grade ≥ 2 acute rectal toxicity using a dosimetric index $D_{25\%}$ and one patient specific variable at a time.

	$log\left(\frac{NTCP}{1-NTCP}\right)\sim$ $D_{25\%}$, age	$log\left(\frac{NTCP}{1-NTCP}\right)\sim$ $D_{25\%}$ diabetes	$log\left(\frac{NTCP}{1-NTCP}\right)\sim$ $D_{25\%}$, PSA	$log\left(\frac{NTCP}{1-NTCP}\right)\sim$ $D_{25\%}$, Statins
α_0	-8.98 [-18.09, -1.23] P=0.035	-5.54 [-10.29, -1.68] P=0.01	-5.45 [-11.08, -0.88] P=0.03	-4.25 [-8.95, -0.32] P=0.05
$D_{25\%}$	0.095 [-0.002, 0.208] P=0.075	0.1 [0.006, 0.217] P=0.05	0.176 [0.052, 0.333] P=0.01	0.087 [-0.011, 0.200] P=0.1
β_1	0.051	1.0	-0.426	-1.28

	[-0.04,0.15]<br>P=0.29	[-0.73, 2.62]<br>P=0.23	[-0.813, -0.142]<br>P=0.01	[-0.266, -0.086]<br>P=0.05
AUC	0.66<br>[0.51, 0.83]	0.68<br>[0.55, 0.81]	0.78<br>[0.68, 0.89]	0.73<br>[0.59, 0.86]

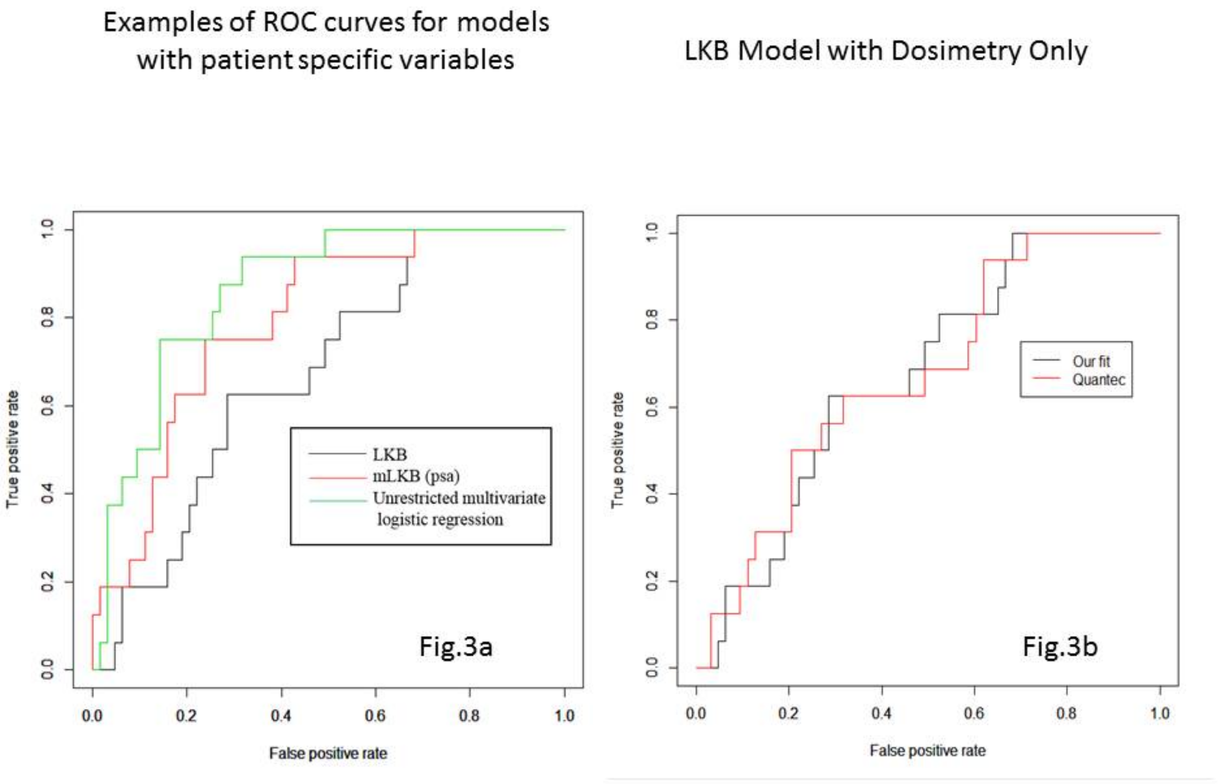

Fig.3

Figure 3: Examples of ROC curves for a subset of models that were discussed in this work. Only a subset of models discussed in the paper is shown to preserve clarity. The mLKB(psa) curve refers to modified LKB model with PSA as a patient specific variable (Table 4). The "Unrestricted Logistic Regression" curve refers to the unrestricted logistic regression model which includes all four patient specific variables that were selected by the LASSO operator, and one dosimetric variable (Table 2). The right panel (Figure3b) shows the LKB model with dosimetry only (black curve) compared to the ROC curve for the QUANTEC late rectal toxicity formula (red curve).
